# Influence of the new mental health legislation in India

**DOI:** 10.1192/bji.2019.18

**Published:** 2020-02

**Authors:** Rakesh K. Chadda

**Affiliations:** Professor and Head, Department of Psychiatry and Chief, National Drug Dependence Treatment Centre, All India Institute of Medical Sciences, New Delhi 110029, India. Email: drrakeshchadda@gmail.com

**Keywords:** India, mental health, legislation

## Abstract

This paper discusses the influence of the Mental Healthcare Act 2017 on mental healthcare in India. The new Act was introduced to meet the recommendations of the United Nations Convention on the Rights of Persons with Disabilities. Reforms proposed in the new legislation, challenges in their implementation and their effects on mental healthcare in the country are further discussed.

India has published new mental health legislation called the Mental Healthcare Act (MHCA) 2017, which came into force from 7 July 2018 and replaced the Mental Health Act (MHA) of 1987. The new mental health legislation was required because the old MHA 1987 was considered insufficient to protect the rights of persons with mental illness in light of the United Nations Convention on the Rights of Persons with Disabilities of 2006.

MHCA 2017 states in its introduction that it is ‘An Act to provide for mental healthcare and services for persons with mental illness and to protect, promote and fulfil the rights of such persons during delivery of mental healthcare and services and for matters connected therewith or incidental thereto’. MHCA specifically aims to protect and promote the rights of persons with mental illness during delivery of healthcare in institutions and in the community, and to ensure mental healthcare, treatment and rehabilitation in the least restrictive environment possible. The Act has provisions for involuntary (supported) admission and treatment of persons with high support needs in mental health institutions, with defined procedures for those needing admission for up to 30 days and those requiring a hospital stay of more than 30 days. Similarly, there is a procedure for admission of minors. All admissions longer than 30 days are to be notified to the designated Mental Health Review Board (MHRB). There is also a provision for emergency treatment for persons with mental illness in a health establishment or in the community. The Act has introduced the concepts of advance directives and nominated representatives; it also includes extensive details about the rights of persons with mental illness and outlines the duties of the appropriate governments in this regard. To implement the new provisions, there are directions to the central and state governments to establish a Central Mental Health Authority, as well as State Mental Health Authorities (SMHAs) at the state level and MHRBs at a district level. As it involves substantial changes from the previous MHA of 1987, the MHCA 2017 is likely to have a major influence on mental healthcare in India. The proposed reforms were in general well received by various stakeholders, according to media reports, but their implementation will not be an easy process, considering the limited resources available in the country.^1^ This paper discusses the effects of the MHCA on mental healthcare in the country.

## New definition of mental illness

The MHCA 2017 provides a comprehensive definition of mental illness as ‘a substantial disorder of thinking, mood, perception, orientation or memory that grossly impairs judgment, behaviour, capacity to recognise reality or ability to meet the ordinary demands of life, mental conditions associated with the abuse of alcohol and drugs, but does not include mental retardation which is a condition of arrested or incomplete development of mind of a person, specially characterised by subnormality of intelligence’. Thus, patients with common mental disorders such as depression, anxiety disorders and even psychoses, where judgement, capacity and ability to meet the ordinary demands of life are not affected, may not come under this definition of mental illness for purposes of the Act and therefore can be admitted to or discharged from a hospital in the same manner as patients with any other illness.

## Regulation of general hospital psychiatric units in the MHCA

One of the major criticisms of the new legislation is that it brings general hospital psychiatric units (GHPUs) under the Act. GHPUs in India have been a unique service provider in the mental health sector, with open psychiatry wards where patients are admitted with a family member who stays with them during the period of admission.^2^ Admission and discharge are on a voluntary basis, with all the patients in the wards staying along with their family members. The patients are not in individual rooms but in halls with capacities varying from six to 20 beds, with family members of all the patients also staying in that accommodation. Such a setting reduces the risk of human rights violations. There have been few reports of human rights violation of persons with mental illness in GHPUs. The GHPUs have visiting hours in the morning as well as evenings. The psychiatry unit is one of various in-patient units in a general hospital and functions like other medical specialities.^3^

The duration of hospital admission in a GHPU is generally short, varying from a few days to a few weeks. Bringing GHPUs under the MHCA is likely to lead to longer hospital stays owing to inclusion of involuntary admissions, thus reducing the availability of active beds, which are often limited to around 20–40 in most of the GHPUs in India. This will affect training in psychiatry, as most undergraduate and postgraduate teaching in psychiatry in India takes place in GHPUs.

The MHCA 2017 stresses that admissions to the mental health establishment should be voluntary (independent) where possible. The procedure for involuntary (supported) admission as stated in the MHCA 2017 is more cumbersome than that in the MHA 1987. The MHA 1987 had a provision for ‘admission under special circumstances’, which permitted admission of a patient on the written request of a family member. This facilitated admission of a large number of patients who were not in a condition to give consent, without going through legal formalities that may lead to delay.^4^

## Protection of the rights of persons with mental illness

One of the biggest contributions of the MHCA 2017 is its significant emphasis on the rights of persons with mental illness, with 11 sections (18–28) devoted to this subject. These include rights to access mental healthcare, community living, protection from cruel, inhuman and degrading treatment, equality and non-discrimination, information, confidentiality, restrictions on release of information in respect of mental illness, access to medical records, personal contacts and communication, legal aid, and how to make complaints about deficiencies in the provision of services. This is a positive development, but the existing mental healthcare resources, including mental health professionals, and out-patient and in-patient services (including those in the private sector) are grossly inadequate to provide for the above-mentioned rights. Budgetary allocation for mental health is less than 1% of the total health budget in India; this needs drastic enhancement if adequate measures are to be taken to ensure that the rights of persons with mental illness as stated above are not violated.^5^ The Act requires a number of initiatives from the government, such as ensuring availability of mental healthcare for all, and community care and residential facilities for persons with mental illness. This is a positive step, especially in the context of a huge mental health gap in the country.

Notably, the MHCA fails to take into account the role of the family caregivers who constitute the predominant informal workforce in mental healthcare in India. Families take care of the day-to-day needs of patients, supervising medication, taking them to hospital for consultation and looking after their financial needs, and are also often the target of behavioural outbursts from the patient.^6^

Considering the local realities, MHCA could have kept a provision for family members to stay with patients during hospital admission (wherever feasible), as has been the practice in GHPUs and also in some psychiatric hospitals. This would be helpful in providing continuity of care following discharge from the hospital and would provide an opportunity for psychoeducation for the family, including learning how to take care of the patient, as well as reducing the risk of violation of patients’ rights.^7^

## Actions needed on the part of the state

MHCA envisages a number of actions on the part of the government of India and various state governments for its implementation. The first step is the formulation of central and state mental authority rules. The government of India established the CMHA, together with MHRB and SMHA rules in May 2018, but SMHAs have not still been constituted in most states. Establishing MHRBs at the district level will be a difficult task. There are insufficient judicial and quasi-judicial officers to chair the board and not enough psychiatrists to be members. Much of India has an overburdened judiciary and a grossly inadequate number of psychiatrists. Yet establishment of SMHAs and MHRBs is essential for implementation of the MHCA.

## Advance directive and nominated representative

Advance directives are legal documents that allow any adult to declare his or her decision about the kind of treatment he or she may be given in the case of development of mental illness. The MHCA has introduced the concepts of advance directives and nominated representatives in mental healthcare in India. The relevance of these concepts and their implementation in the country is questionable. With a huge population of more than 1.3 billion, keeping records of advance directives represents a Herculean task. Even the content of advance directives as stated in the Act will be difficult to implement, as there are limited mental healthcare facilities and an overburdened judiciary in the public sector.^8^ Only the MHRBs have the power to amend or overrule an advance directive. There are also issues of practicality. For instance, what happens if a patient has opted for treatment in a private or corporate hospital in their advance directive, but the family cannot afford this – who is going to finance the treatment? Globally, there are limited data to support the use of advance treatment directives in persons with severe mental illness.^9^ Similarly, the concept of a patient's nominated representative may not be appropriate in many cases. The process of applying for and taking responsibility for the patient's (involuntary) admission to a psychiatric hospital against their stated wishes may lead to resentment, anger and even vengeance toward the nominated representative and make families a direct target of patients’ anger and resentment.^10^

## Decriminalisation of suicide

Another important reform brought by the MHCA is decriminalisation of suicide attempts. Until recently, attempted suicide was punishable under Section 309 of the Indian Penal Code. MHCA states that any person who attempts to commit suicide will be presumed, unless proved otherwise, to be suffering from severe stress, and the government will have a duty to provide care, treatment and rehabilitation for such a person to reduce recurrence of the event.

## Conclusion

MHCA 2017 provides for an ambitious and progressive legislation, but it has to be implemented in the context of limited mental healthcare resources. The Act provides for a period of 10 years from its commencement for the government and other stakeholders to meet internationally accepted guidelines for human resources development, hence acknowledging the difficulties of implementation given the huge population of India. Currently, the MHA 1987 cannot be fully implemented because of limited resources, and the MHCA has been introduced without any obvious plan to address how meeting those resource needs will be achieved.
